# A longitudinal faculty development program: supporting a culture of teaching

**DOI:** 10.1186/s12909-019-1832-3

**Published:** 2019-11-01

**Authors:** Annette Burgess, Elie Matar, Brendon Neuen, Greg J. Fox

**Affiliations:** 1 University of Sydney School of Medicine - Education Office, Faculty of Medicine and Health, University of Sydney, Edward Ford Building A27, Sydney, NSW 2006 Australia; 2 Sydney Health Education Research Network (SHERN), Faculty of Medicine and Health, University of Sydney, Sydney, NSW 2006 Australia; 3 Brain and Mind Centre, Faculty of Medicine and Health, University of Sydney, Sydney, NSW 2006 Australia; 4 The Central Clinical School, University of Sydney School of Medicine, Faculty of Medicine and Health, The University of Sydney, Sydney, NSW 2006 Australia; 5 Renal and Metabolic Division, The George Institute for Global Health, UNSW Sydney, Newtown, NSW 2006 Australia; 6 The George Institute for Global Health, PO Box M201, Missenden Rd, Newtown, NSW 2050 Australia; 7 Royal Prince Alfred Hospital, Camperdown, The University of Sydney, Rm 5216, Level 2 Medical Foundation Building K25, 92-94 Parramatta Road, Sydney, NSW 2006 Australia

## Abstract

**Background:**

Recent trends in faculty development demonstrate a shift from short term to long-term programs; formal to informal learning in the workplace; individual to group settings; and from individual support to institutional support. The purpose of this study was to develop and evaluate a one-year Clinical Teaching Fellowship (CTF) program designed to equip early career medical practitioners and basic scientists with necessary skills to facilitate Team-based learning (TBL).

**Methods:**

The CTF program provided formal training, a choice of informal professional development activities, and practical co-teaching opportunities in TBL. Of the 40 registrants, 31 (78%) completed the program. Data were collected via questionnaire and focus group. Data were analysed using descriptive statistics and framework analysis.

**Results:**

Participants considered the CTF program as relevant to their needs and useful to their career. Learning was enriched through the combination of training, practical teaching experience alongside senior clinical teachers, the multi-disciplinary context of training and co-teaching in TBLs; and the sense of community. Competing clinical responsibilities made it difficult to attend training and TBL teaching.

**Conclusions:**

The CTF program provided a longitudinal faculty development framework promoting preparation, practice and development of teaching skills. Securing institutional support to invest in the growth and development of early career teachers is essential to sustained innovation and excellence in teaching.

## Background

Faculty development within healthcare education refers to the activities in which staff participate to improve their knowledge and skills as teachers, educators, leaders, managers, researchers, and scholars [1]. Within the past decade, the number of faculty development programs offered in medical education has increased significantly. This results from changing trends in teaching and assessment methods, and has led to the implementation of a variety of faculty development programs designed to upskill healthcare educators [1]. Although faculty development has traditionally been implemented through formal programs [2], more recently, it has been suggested that knowledge and skills are better developed through informal learning opportunities that take place in authentic environments [3, 4]. The literature indicates that through participation in a community of practice that includes experience, observation, reflection, feedback, and workplace learning, staff develop specific expertise [1]. O’Sullivan & Irby (2014) posit that a faculty development community should embrace the participants, the curriculum, and the workplace context [5].

Medical practitioners and basic scientists play an important role in educating the next generation of medical practitioners. A recent systematic review of faculty development programs within medicine found that formal, structured activities in group settings are most common [6]. These provided a high level of satisfaction among participants; a positive change in attitudes towards teaching, self-reported gains in knowledge, skills and teaching behaviours, and some observed changes in teaching behaviours [6]. However, few changes were reported in organisational practice and student learning [6]. In order to enhance the outcomes of faculty development programs, healthcare institutions need to focus on shifting from short term to long-term programs; from formal to informal learning in the workplace; from individual to group settings; and from individual support to institutional support [7].

In 2017, the first and last authors (AB and GF) developed and implemented a new “Clinical Teaching Fellowship” (CTF) program at the University of Sydney School of Medicine. The program was introduced to transfer theory to practice and achieve a sustainable, scalable faculty development program. It was designed to align with the introduction of Team-based learning (TBL), as a new method of teaching within Years 1 and 2 of the post-graduate medical program [8]. With an annual Year 1 student intake of approximately 300 students, we were aware that the introduction and ongoing teaching support for TBL would require a large number of skilled facilitators. We sought to develop a program that would promote teaching opportunities, and provide support for junior medical staff and basic scientists interested in developing formal skills in medical education. The inaugural CTF program (2017) attracted 24 participants. The second iteration of the program (2018) attracted 40 participants, and two of the CTF 2017 alumni, the second and third authors (EM and BN) joined as co-leads of the program. In 2019, we have received more than 150 applications. The focus of this study is the second iteration of the CTF program, in 2018. The second iteration was chosen, since ethics approval was received for the 2018 study and at the time of publication, the 2019 iteration was not completed.

### Theoretical concept

Theories informing educational practice offer valuable lenses to analyse learning [9]. Dornan and colleagues (2014) proposed the Experienced Based Learning (ExBL) model for workplace education [10]. ExBL acknowledges that an integrated approach that orientates newcomers to the cultural and social aspects of teaching in the medical curricula is required, including meaningful opportunities to participate. It is only through participation that new practices are learnt, and new tasks are progressively undertaken [10].

The ExBL model suggests that learning outcomes are acquired through provision of a supportive learning environment, and participation in authentic workplace activities. This model provides a transferrable blueprint for education that is applicable to the faculty development context [10]. According to the ExBL model, learners’ development in skills and knowledge, is fostered by three key areas of support:

1. **Organisational support**: to ensure the learning experience matches the desired outcomes, and provides opportunities for workplace practice.
2. **Pedagogic support:** provided by teachers in the workplace setting, including mentors, role models and supervisors.
3. **Affective support:** provided by a warm and inclusive environment.

The purpose of our study was to develop, implement and evaluate a longitudinal (1 year) Clinical Teaching Fellowship (CTF) program for junior medical staff and basic scientists. Further, to explore participants’ perceptions of the structure, processes and outcomes of the program, utilising the conceptual framework of ExBL.

## Methods

### Course design

The CTF program was largely designed to prepare medical practitioners and basic scientists, for their new roles as skilled TBL facilitators; and potential future leaders in medical education. A coherent and flexible platform of activities was provided, that built upon each other to make use of various instructional formats, and to fit the personal needs of participants. The learning activities served as exemplars of modern learning methodologies.

They were defined based upon the specific skills required by the medical program, and aligned with the educator’s interest.

**Fig. 1:**
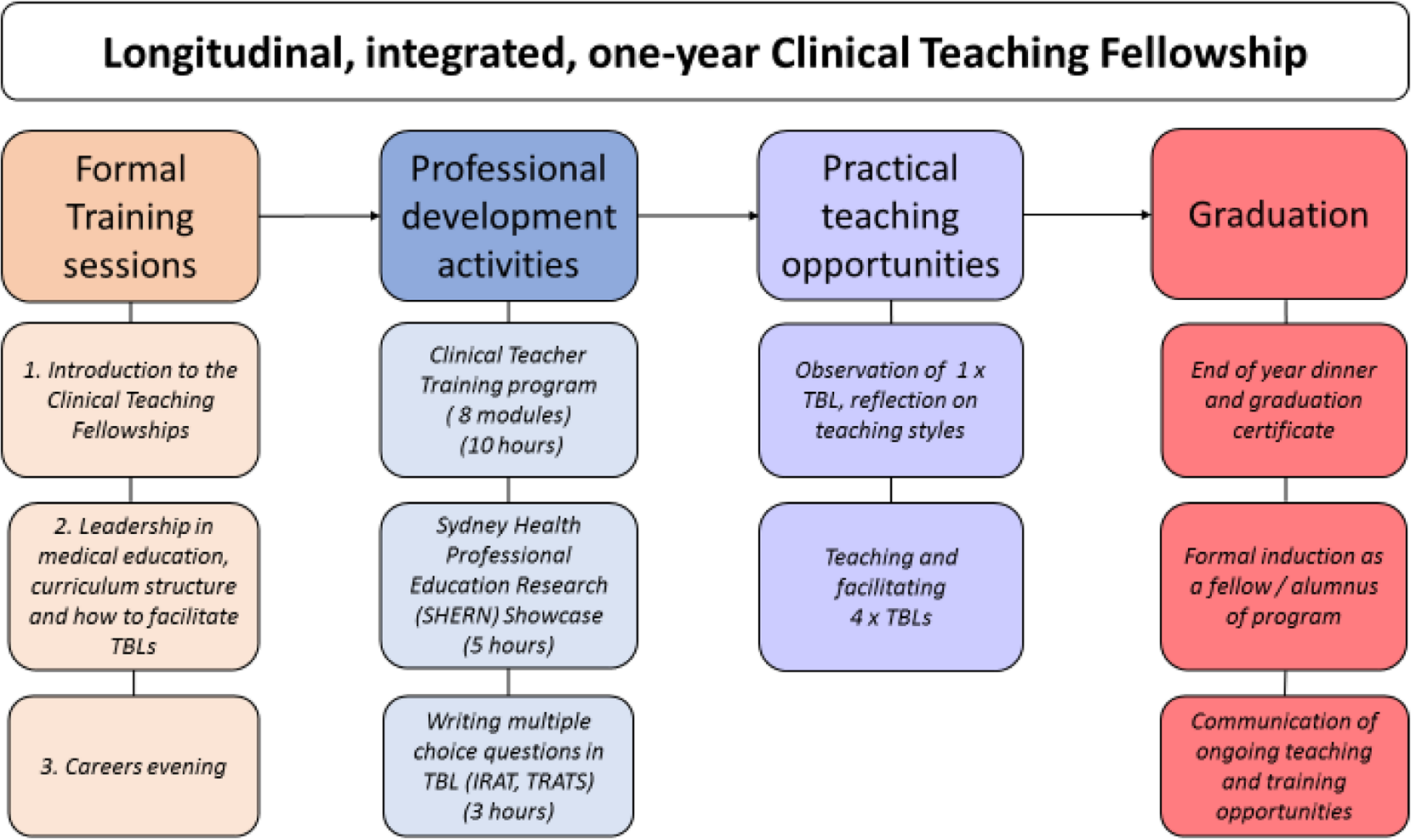
Integrated function of the one-year Clinical Teaching Fellowship program

Mandatory, formal requirements to succeesfully complete the CTF program included attendance at two TBL teaching and leadership training (faculty development) sessions, observation of one TBL class; and co-facilitation of four TBL classes. Each TBL class was 2.5 h in duration. Most of the training was run in TBL format, providing a scalable model for future implementation. Optional, informal activities included participation in: the Clinical Teacher Training program [11]; careers evenings and networking dinners featuring guest speakers with senior roles in medical education; a Multiple Choice Writing session; and Medical Education Research meetings. Most of these sessions were offered at various dates and times, including evening sessions, to provide flexible arrangements, and encourage attendance. All practical teaching activities were allocated for the CTFs, that is, they were not required to actively seek teaching or professional development opportunities, rather, this was organised for the CTFs in accordance with their preferences. These formal training sessions and optional activities took place throughout the year, culminating in a graduation dinner as a formal induction into an enduring community of practice. A summary of the training, teaching and professional development activities is shown in Fig. 1.

### Study design

#### Recruitment

Early career medical staff (resident medical officers, specialty trainees, and recently qualified consultant physicians) at teaching hospitals affiliated with the University of Sydney School of Medicine; and basic scientists (graduate students, post-doctoral researchers and early career researchers) from associated medical research institutions were invited by email to participate in the CTF program. Our aim in recruitment was to be inclusive, rather than targeting those who had already taught in the medical program.

### Data collection and analysis

#### Quantitative data

Quantitative data were collected from participants by a post-program questionnaire, reflecting on participants’ perception of:

- the content of the CTF program, and the training provided
- TBL facilitation experience
- networking opportunities
- intention to participate in future student teaching activities; and
- intention to participate in future CTF program activities

Participants were asked to respond to 12 closed items, using a five-point Likert scale ranging from ‘strongly disagree’ (1) to ‘strongly agree’ (5). Data were analysed using descriptive statistics.

#### Qualitative data

Qualitative data were collected via questionnaire and focus group. The questionnaire contained two opened ended questions: “What did you find to be the most useful aspects of the Clinical Teaching Fellowship program?” and “What suggestions would you make for improvement to the Clinical Teaching Fellowship program?”. Additionally, at the end of the final face-to-face session, all participants were invited to a focus group. The focus group was recorded and transcribed verbatim. Following consultation with all authors, the first author (AB), used framework analysis [12] to code the data set, including the qualitative feedback from the questionnaire, and the focus group, using the ExBL as a conceptual framework [10].

### Ethics approval

The University of Sydney Human Research Ethics Committee approved the study: project number: 2018/685.

## Results

### Registration and demographics

In 2018, 30 medical practitioners and 10 basic scientists registered for the CTF program. Of the participants, 17 were male and 23 female. The medical practitioners included: 25 speciality trainees, three consultant physicians, and two consultant general practitioners. Participants were based across 10 metropolitan teaching hospitals and affiliated institutions. Thirty one out of 40 (78%) participants completed the CTF program. Of these 31 participants, six were basic scientists, and 25 were medical practitioners. In total, the 31 CTFs provided approximately 372 h of voluntary face-to-face teaching, an average of 12 h per participant.

### Quantitative data

**Fig. 2:**
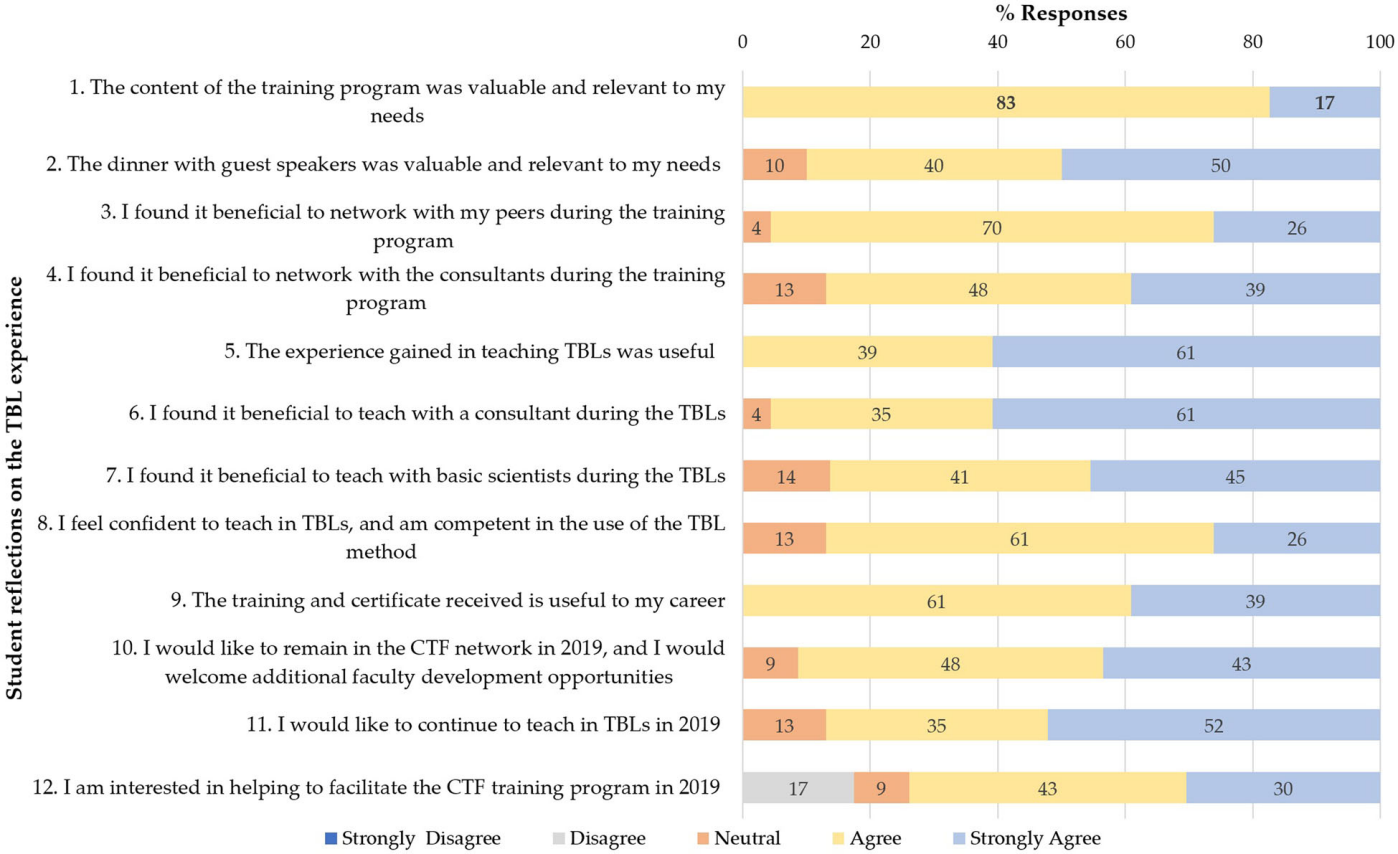
Clinical Teaching Fellowship (CTF) participant experience (*N* = 23)

Following the graduation dinner, 23/31 (74%) of participants completed the questionnaire. Participant responses to the quantitative items on the questionnaire are shown in Fig. 2.

### Qualitative data

Four participants (13%) attended the focus group session: three medical practitioners; and one basic scientist. Combined qualitative data from the responses to open-ended questions on the questionnaire (completed by 23/31 (74%) of participants) and the focus group are presented in Tables 1, 2, and 3. The results are presented using the conceptual framework of Experienced Based Learning model [10].

**Table 1:** Participants’ responses regarding their perceptions of their experiences that related to ‘Organisational support’

Organisational support:	Learning experience sits appropriately within the curriculum, with opportunities to participate in practice.
Formal training Participants found the formal training prior to TBL classes, and professional development activities throughout the year, a valuable component of the CTF program	*“I thought that was a really good set up on the first night of when they went through everything. I felt confident going into it”.* *“Training in TBLs. Hearing from people who have careers in medical education, role play and teaching techniques, visiting speakers”.*
Preparation and Resources Participants felt they were provided with support and the resources needed in preparation for the TBL classes.	*“The facilitators had a lot of support going into the program with all the resources we had a very clear layout of how it was going to be, and the class was planned out. It was a lot of work (preparing), but it pays off on the day”.*
Learning methods in workshops Participants felt supported with the training that was provided on different pedagogies within education.	“*I enjoyed my session where we taught each other using different teaching techniques”* *Learning about the pedagogy of TBL, and other teaching methods …*. *Learning a new model of delivering medical education”.*
Mix of formal and informal training and professional development activities Participants found it beneficial to have a mix of formal and training sessions, and practice in TBLs. However, some participants felt an online component of the program would provide a valuable addition to the program.	*“It was a nice mix of formal learning about teaching methods and having the opportunity to be involved with TBLs and observe how they run and to work with people who’ve done it before”.* *“I think these kind of nights of having the mentorship aspect was really valuable and even informally. And then actually doing the TBL sessions was quite rewarding”.* *“Having the formal training nights where you get to hear people who have gone before in that path, and take their advice, and just meeting other fellows as well, that’s really helpful”.* *“It would be useful to have some way to reiterate what we’re learning into how we access this program. So being able to go online, and answer some questions about it afterwards to demonstrate what we learnt”.*
Content revision Participants found that the revision of content required to teach TBLs was relevant to their own learning. The clinicians revised the basic science knowledge, and the basic scientists learnt how their knowledge was applied to a clinical context.	*“It was useful to revise topics (basic science) relevant to my own learning and provide a clinical context to students”.* *“Refreshing my knowledge of the science underpinning the clinical aspects”.*
Structure and formality of the CTF program The structure and formality of the CTF program provided links to junior clinical staff and basic scientist that might not otherwise exist in the large teaching hospitals. Participants valued the formal recognition the CTF program provided, as well as recognition of their contributions to teaching.	*“The teaching opportunities can get lost at big hospitals, so it is nice to have the opportunity presented to you”.* *“I think being able to put something like that on your CV for an academic CV is quite useful, so it’s a lot more recognition that you just doing teaching”.* *“The acknowledgement of teaching contributions in TBLs by way of a formalised program.”*

**Table 2:** Participants’ responses regarding their perceptions of their experiences that related to ‘Pedagogic support’

Pedagogic support	Pedagogic support is provided by teachers in the workplace setting, including mentors, supervisors, role models, as well as sources of informal support.
Team-teaching design of TBL Participants valued the team teaching design, where co-facilitation in TBLs occurred with different disciplines and levels of training. They also appreciated the TBL design itself, where they could practice teaching to both large and small groups of students.	*“It was very valuable to have all the different registrars and consultants and basic scientists, all working together and talking to the whole room of students, but then also having the chance to go around and talk to the individual small groups of students, I thought that was interesting and rewarding”.* *“Variety of backgrounds were useful when answering questions.”*
Observation of TBL CTFs appreciated the opportunity to observe a TBL class, discuss the teaching methods with a senior teacher, and reflect on their experience, prior to teaching the TBL themselves	*“I found observing a TBL invaluable because I had never had anything to do with TBLs before, so it was really good to see how they worked, and also just to be able to ask a few questions of the facilitators. One facilitator was very helpful, at the end he led a troubleshooting discussion – for example, what would you do if one student is dominating the discussion, or they’re not thinking very deeply”.*
Development of their own teaching style CTFs felt that by co-teaching, they were able to adjust to the structured format of TBL, and that teaching with others assisted in development of their own teaching styles. The felt supported during the class by co-facilitating with senior clinicians or basic scientists	*“Developing skills and confidence in teaching a large group. Developing an individual teaching style while teaching and learning with consultants”.* *“Getting used to the TBL structure by watching other teacher’s teaching style”.*
Role modelling of senior teachers Participants valued the opportunity to teach with and learn from senior clinicians	*“Working with senior clinicians gave me invaluable teaching insights”.* *“It was very helpful to have the opportunity to facilitate with senior scientists and clinicians, in order to learn different styles of teaching”.* *“I though the combination of having the experience where you’re actually doing the teaching with a body of people that have done it before, that’s been really handy”.*
Multidisciplinary aspect during training and TBL facilitation Both clinicians and basic scientists found the content and context provided by each other’s discipline to be of value.	*“Attending the workshops and the TBL teaching in collaboration with basic scientists and clinical specialists”.* *“From a basic scientists view, it was very interesting and useful for me to get the clinical perspective.”* *“Super valuable to have the basic scientists and clinicians in each TBL class. It brings something completely different. I think the senior clinicians have a global perspective, and the basic scientist goes into the minutia of whatever, and really answer the students’ questions, and the registrar provide a near peer teaching element, which is useful because they understand best what the medical students know because we’ve only just done it, really – four to six years ago – you still remember what it’s like.. and what you didn’t understand and why. It is useful to have all three involved. We had a number of times where one of us was asked a question, and we could help each other”.*
Provision of Feedback Some participants indicated that a greater amount of feedback was needed in order to guide improvement of teaching skills	*“I would like to receive more feedback as to your performance at teaching TBL’s”.*

**Table 3:** Participants’ responses regarding their perceptions of their experiences that related to ‘Affective support’

Affective support	Affective support is provided by a warm and inclusive learning environment
Networking Participants appreciated the networking opportunities. However, they indicated that although they would like to continue teaching, their movement around different clinical schools and hospitals may make it difficult to stay engaged as alumni.	*“Sense of community and mentorship., including the basic principles of TBL, and teaching and was taught, lectures, exposure to TBL”.* *“opportunity to work with fellow doctors”* *“I enjoyed the teaching and networking opportunities. I would like to stay involved in the program in 2019 but will largely be based in a different hospital network”.*
Opportunity to interact with students Participants welcomed the chance to interact with students, and learn from teaching students.	*“I enjoyed the teaching experience and interactions with students. I learnt far more than I contributed”.* *“Opportunity to teach medical student and interactions with them”.*
Training and professional development events Participants valued meeting fellow CTF’s at training evenings. CTFs found it motivating to meet others who were enthusiastic about teaching	*“I think there’s a real value to being here in person as well because that’s where you meet everyone, and get to have conversations with people”.* *“Seeing that there’s people out there who are passionate about teaching is always useful and where n you’re amongst such a bunch of people that’s encouraging”.*
Larger network needed/work commitments/Difficult getting to TBLs A larger support network is needed to work across hospital sites and engage Fellows in teaching activities that fit within their work environment	*“The thing I found tricky was having the opportunity to attend TBLs, I had exam preparation for basic physicians exams. I often had critically unwell patients in the mornings, and there was no cover, that was tricker than I anticipated. Also, I was rotated out to a country rotation. It would be good if there is some way to organise tutorials to do with students when their rural settings …*. *and you could bring those experiences back”.* *“It has to be done around clinical service … there needs to be a formal way of covering for people who are attending TBLs because otherwise it’s impossible to negotiate”.* *“It can be quite challenging to attend the whole TBL session as a ward registrar. Would it be feasible to have the option to attend half of the session, and assist in TBL development in other ways?”* *“It would be very useful if this is built into basic physician contracts... and say “well, actually I’m doing a teaching role so this many hours of the week needs to be dedicated to this”.*

The theme of ‘Organisational support’ is illustrated in Table 1. Participants regarded the formal training prior to TBL classes, and professional development activities throughout the year, to be valuable components of the CTF program. They felt structure and formality of the CTF program provided links to junior clinical staff and basic scientists that are lacking in large teaching hospitals. Participants felt they were provided with support and the resources needed in preparation for the TBL classes. They found it beneficial to have a mix of formal and training sessions, and practice in TBLs. However, some participants felt an online component of the program would provide a valuable addition to the program. Participants valued the formal recognition of their contributions to teaching that the CTF program provided.

The theme of ‘Pedagogic support’ is illustrated in Table 2. Participants valued the team teaching design, where co-facilitation in TBLs occurred with different disciplines and varied levels of training. Both clinicians and basic scientists found the content and context provided by each other’s discipline to be of value. They also appreciated the TBL design itself, where they could practice teaching to both large and small groups of students. Participants appreciated the opportunity to observe a TBL class, discuss the teaching methods with a senior teacher, and reflect on their experience, prior to teaching the TBL themselves. While most appreciated the feedback provided after their teaching session, some participants indicated a need for greater feedback.

The theme of ‘Affective support’ is illustrated in Table 3. Participants appreciated the networking opportunities provided by the CTF program, and found it motivating to meet others enthusiastic about teaching at training and professional development activities. However, they indicated that although they would like to continue teaching, their movement around different clinical schools and hospitals (regulated by their working arrangements), may make it difficult to stay engaged as alumni. They felt a larger support network is needed to work across hospital sites and engage participants in teaching activities that fit within their changing work environments.

## Discussion

This study sought to explore participants’ perceptions of a new longitudinal Clinical Teaching Fellowship (CTF) program, specifically designed to support the professional development of participants’ skills in TBL facilitation, and their engagement in medical education. The vast majority of participants regarded the training and experience provided to be relevant to their needs and beneficial to their career. Participants felt their learning was enriched through a combination of formal training, practical teaching experience alongside senior clinical staff, the multi-disciplinary context of training and co-teaching in TBLs; and the ‘sense of community and mentorship’ provided through the CTF program. Most expressed a desire to continue teaching TBLs in future years, and an interest in remaining connected with the CTF program. Some participants felt there should be a greater emphasis placed on provision of feedback on their TBL teaching. Additionally, participants indicated that clinical responsibilities made it difficult to attend formal training and teaching sessions. These findings are expanded and discussed using the conceptual framework of ExBL.

### Organisational support

Organisational support ensures that the learners’ experiences are aligned with the program outcomes, and that there are opportunities for active participation [10]. Learning is context-dependent, and requires appropriate opportunities to practice what has been learnt [13]. Engagement in the CTF program was achieved through provision of formal training and structured opportunities to gain relevant practical experience. By having the practical teaching experience and professional development carefully organised for the participants, in accordance with their preferences, the participants felt supported by staff in their new teaching roles. Participant preferences for teaching and professional development activities were decided by participants according to their individual expertise, interest and availability. This type of structured and organised support is emphasised by Dornan and colleagues as essential to ensuring learner engagement at the appropriate level [10]. Although the medical practitioners and basic scientists held a variety of roles within their respective institutions (university, hospitals, clinical schools, research institutes), a clear majority of participants felt the training they received was relevant to their TBL teaching endeavours. Participants appreciated being afforded the opportunity to take part in a structured program, with a formal certificate highlighting their commitment to medical education and professional development.

### Pedagogic support

Pedagogic support refers to the support that is provided by the teachers in the workplace environment [10]. The social congruence of participants, and the opportunity to network with each other at training sessions afforded a richness to their learning. Participants were able to draw from the experience of others, both during training and teaching sessions. During co-teaching in the TBLs, the participants were dependent upon each other’s knowledge and skills, relating to either basic sciences, or clinical competencies. Teaching skills were largely socially constructed [14], through observation, experience in co-teaching, and provision of feedback by senior co-teachers. However, some CTFs expressed a desire for greater feedback on their TBL facilitation. It is widely recognised within medical education that teachers feel they give more feedback than learners claim they receive [15]. Although frequently reported as lacking, observation with feedback provides a powerful means to improve skills and change the behaviour of learners [15]. This will be an important area for development in future iterations of the CTF program.

The CTF program emphasised the professional expectations of the basic scientists and medical practitioners as teachers. This focus on professional identity formation is relevant in contemporary faculty development programs, particularly for early-career staff who may hold a number of different roles. Steinert et al. (2019) state that clinicians and basic scientists often feel that they are undervalued by their institutions [16]. They suggest that ‘bolstering’ their identities as teachers through faculty development programs may increase engagement in educational activities, and provide a ‘sense of community’. [16] Participants commented that they found interactions during co-teaching of TBLs valuable, with junior and senior staff from across disciplines sharing experiences. The CTF role as a TBL facilitator required meaningful preparation, and responsibilities in teaching were aligned with the Clinical Teaching Fellows’ abilities, and competence was developed.

### Affective support

Affective support is provided by a warm and inclusive environment, where participants feel they are treated as a member of one large community [10] and where similar goals and a sense of belonging is fostered [17]. The actions of supervisors, role models and mentors in the workplace are important in shaping the experience and behaviours of trainees [13]. Participants felt they learnt from their co-facilitators, and also from their interactions with students during the TBL classes. CTFs were able to develop their teaching skills, and were also prompted to revise their own content knowledge. However, it is important to note that 9/40 (23%) of initial CTF registrants did not manage to complete the minimum requirements of the program. Indeed, even among those who did complete the program, some of the clinicians expressed concern around their availability to attend TBLs (which are held during working hours) due to clinical service requirements. Protected teaching time, and incorporating additional structured teaching activities based in participants’ home institutions as part of the CTF program may help to address this issue in the future. It is anticipated that a decentralisation of the initial training and teaching activities, will improve accessibility of opportunities, and therefore completion rates. In 2019, in an attempt to improve CTF completion rates, we will offer additional optional professional development activities at various hospital sites in order to offer further flexibility, especially for busy clinicians.

## Limitations

This was a pilot study with a small sample size, and the opinions of participants may not be generalisable to all clinical educators in similar settings. We note that our response rate to the questionnaire was at 74%, and only 13% of participants took part in the focus group. Therefore, the responses may not be representative of the entire group of CTFs. There are challenges of aligning participant and institutional goals. However, the faculty development program is applicable to non-TBL settings. Implementation of the CTF program required institutional leadership support at a senior level.

## Conclusion

The CTF program demonstrated the feasibility and acceptability of a longitudinal faculty development framework within medical education, that is also applicable to non-TBL settings. The program allowed participants to prepare, practice and improve their teaching skills, within a supportive environment. In 2019, the number of CTF applications has continued to grow, with an increase in applications of 350% compared to the second year. Our findings suggest that through provision of opportunities for delivery of theory, choices in professional development activities, workplace practice in teaching with supportive co-teachers, and alumni engagement, a sustainable community of practice can be built. Securing institutional investment to support the growth and development of our volunteer teachers will be essential to ensure sustained innovation and excellence in clinical teaching.

## Data Availability

Datasets supporting the conclusions of this article are included within the article. Additional data at the level of individual participant is not available as per confidentiality agreements approved by the Human Research Ethics Committee, University of Sydney.

## Abbreviations

CTF: Clinical Teaching Fellowship
TBL: Team-based learning
